# Changes in young adults' mental well-being before and during the early stage of the COVID-19 pandemic: disparities between ethnic groups in Germany

**DOI:** 10.1186/s13034-021-00418-x

**Published:** 2021-11-23

**Authors:** Stephanie Plenty, Chloe Bracegirdle, Jörg Dollmann, Olivia Spiegler

**Affiliations:** 1 Swedish Institute for Social Research, Stockholm University, 106 91 Stockholm, Sweden; 2 Institute for Futures Studies, Box 591, 101 31 Stockholm, Sweden; 3 Nuffield College, University of Oxford, Oxford, UK; 4 Mannheim Centre for European Social Research, University of Mannheim, Mannheim, Germany; 5 German Centre for Integration and Migration Research (DeZIM), Berlin, Germany

**Keywords:** Mental well-being, Ethnic/racial minority groups, Young adults, COVID-19, Stressors, Depression, Anxiety, Life satisfaction, Psychosomatic complaints, Discrimination

## Abstract

**Background:**

The COVID-19 pandemic resulted in substantial disruptions to the daily lives of young people. Yet knowledge is lacking about changes in mental well-being among young adults, whether those from ethnic minorities were more adversely impacted by the pandemic than the ethnic majority, and the extent to which pandemic-related stressors contributed to any declines in mental well-being.

**Methods:**

We draw on nationally representative German CILS4COVID data, collected early in the pandemic (*N* = 3517, M_age_ = 25). Respondents provided information on mental well-being (psychosomatic complaints, anxiety, depression, life satisfaction) and exposure to pandemic-related stressors (financial worries, health worries, discrimination, contact with COVID-19). Responses on mental well-being were matched to responses from two pre-pandemic waves. Individual fixed effects regressions examined ethnic group differences in changes in mental well-being prior to, and at the early stage of, the pandemic. Path analysis tested the role of pandemic-related stressors in declines in mental well-being.

**Results:**

Overall, young adults’ mental well-being had improved at the pandemic assessment compared to pre-pandemic assessments, and few ethnic group differences in changes were found. However, greater pandemic-related stressors were associated with worsened mental well-being at the pandemic assessment. Among Asian minorities, indirect effects were found on anxiety via health worries, and on depression via health worries and discrimination. For Turkish, Middle Eastern and African minorities, indirect effects on anxiety and depression were found via health worries.

**Conclusions:**

We did not find widespread declines in mental well-being among young adults at the early stage of the pandemic, and changes in mental well-being prior to and at the early stage of the pandemic were mostly similar across ethnic German and minority groups. Nevertheless, pandemic-related stressors posed risks for young adults’ mental well-being, particularly increased discrimination and health worries among Asian minorities, and health worries among Turkish, Middle Eastern and African minorities.

**Supplementary Information:**

The online version contains supplementary material available at 10.1186/s13034-021-00418-x.

## Introduction

There are concerns about the negative impact of the COVID-19 pandemic on young people’s mental well-being, particularly those in already vulnerable populations. Young adulthood is a period when transitions, uncertainties and mental health difficulties are common, and for many young adults the pandemic instigated major disruptions to daily life. At the same time, immigrant and ethnic minority communities bore a disproportionate brunt of the pandemic’s health, social and economic burdens [27, 36], which may present additional challenges to the mental well-being of young adults in these groups. However, there is a lack of empirical evidence on changes in young adults’ mental well-being prior to and after the onset of the pandemic, and how or why changes may have differed between young adults from ethnic minority and majority groups. Such evidence is required to inform strategies to assist young adults in emerging from the pandemic successfully and to minimise exacerbating existing inequalities.

### Young adults and mental well-being during the COVID-19 pandemic

Young adults, or so-called emerging adults (ages 18 to 25 years) [1], are a relatively new population of interest, falling at the intersection of the more easily defined populations of adolescents and adults. The young adult period is typically characterised by transitions and rapid development in multiple domains, including work, study, living arrangements as well interpersonal and romantic relationships [1, 41], which are often experienced as stressful [34]. The introduction of the COVID-19 mitigation measures in early 2020 meant that many young adults became unemployed or had their work hours reduced [3], and those studying were required to swiftly adapt to online learning. Social support, networking and career building opportunities were heavily reduced during a life period when young people are striving to establish themselves in various aspects of adult life. Furthermore, well before the COVID-19 pandemic, young adults in Europe and Northern America were already at greater risk of poorer mental well-being than older adults. Rates typically increase from mid-adolescence until approximately 20 years of age before starting to improve towards the thirties [18, 22, 46], although the precise age and rate of change for improvements is unclear. Thus, the pandemic may have compounded existing stressors or introduced additional stressors that reduced young adults’ mental well-being.

Following the onset of the pandemic, younger adults were reported to have a higher prevalence of mental health problems than older adults (18–34 years in [4], 18 to 30 years in [20], 18–29 years in [30]). Studies on university students found within-person declines in mental well-being compared to one year before the pandemic (stress, anxiety, loneliness, and depressive symptoms among undergraduate students in [17]) and across the initial months of the pandemic (stress and anxiety among 18–22-year-olds in [21]), as well as declines compared to earlier cohorts (graduate students in [10]). Examining within-person changes among nationally representative samples, two UK studies found a deterioration in mental health among adults from pre-pandemic (2017–2019) to pandemic (April 2020) assessments that was strongest among young adults (18–34 years in [13], 18–24 years in [37]). However, not all studies have observed a clear decline in mental well-being among young adults. For example, a U.S. study found no overall change in psychological distress among adults between February 2019 and May 2020, yet it did find that when increases occurred, they were more likely among those aged 20–39 years than older adults [5]. Furthermore, although a UK study found that rates of anxiety and general poor mental well-being were higher among young adults (aged 28 years) in April–May 2020 compared to pre-pandemic assessments two to four years earlier, depression was less prevalent during the pandemic assessment [26]. Thus, although most studies point to a vulnerability in young adults’ mental well-being early in the pandemic, tests of changes compared to pre-pandemic levels are rare and have produced mixed findings.

### Minority groups and pandemic-related stressors

To date, investigations of young adults’ mental well-being after the onset of the pandemic have overlooked the potentially vulnerable social position of young adults from ethnic minorities compared to those of the majority population. Yet, there have been reports of increased ethnic discrimination (e.g., [28]), presumably related to greater feelings of threat and insecurity triggered by the pandemic. During the early stage of the pandemic, ethnic discrimination was primarily observed towards individuals of Asian descent [15, 28], likely due to the initial identification of the virus in Wuhan, China. In addition, given that racial and ethnic minority group members are more likely to contract COVID-19 than majority group members [9, 27], contact with COVID-19 and worries about one’s health may also be more common among young adults from minority groups. Furthermore, young adults of immigrant origin are less likely to be in secure study situations than the majority population [38], and the employment situation of disadvantaged minority groups has been heavily hit by the pandemic [36]. Thus, young adults from ethnic minorities may experience greater financial worries than young adults from the ethnic majority. During the early stage of the pandemic in Germany, Soiné et al. [42] found greater health and financial worries among young adults (24–26 year olds) with former Yugoslavian or Turkish backgrounds compared to those with a German background, which is consistent with the disadvantaged socioeconomic positioning often observed among these minority groups. Greater health worries, but not financial worries, were also found among young adults with an Asian background.

It appears that individuals from minority groups are more likely to have experienced pandemic-related stressors of ethnic discrimination, exposure to COVID-19, and worries than individuals from ethnic majorities. As each of these risk factors are associated with poorer mental health (e.g., depression, anxiety, stress, PTSD) [8, 28, 29, 40], greater declines in mental well-being among young adults from ethnic minorities compared to the ethnic majority could be expected. Cross-sectional studies have reported worse mental well-being among young adults (aged 16 to 24 years in Dewa et al. [14]) and adults [20] from immigrant and ethnic minority groups compared to the majority population during the pandemic. However, longitudinal studies among adults have found few group differences in decreases in mental well-being between pre-pandemic (2017–2019) and pandemic (April 2020) assessments [5, 39, 45]. Importantly however, no studies have examined changes among young adults specifically, leaving the question of whether the pandemic has had a more detrimental impact on the mental well-being of young adults from minority groups unaddressed.

### Methodological limitations of previous research

Current knowledge on the mental well-being of young adults during the early stage of the pandemic suffers from three key methodological limitations. Firstly, many studies are cross-sectional, meaning that group differences may reflect pre-existing problems and inequalities rather than pandemic-related changes and disparities. Some studies have compared mental well-being during the pandemic to previous cohorts. However, given the international trend in recent years of increasing psychological distress among young people [44], cohort comparisons may overestimate the negative effects of the pandemic. Secondly, longitudinal studies tracking the same individuals either follow participants only within the pandemic or from one pre-pandemic time-point ([26] is an exception). While these studies have many benefits, they preclude an understanding of changes in mental well-being prior to the pandemic, which is necessary to clarify if changes are unique to the pandemic or part of pre-existing trends. Thirdly, much current knowledge on young people is based on findings from small and unrepresentative samples. Many studies have employed nonprobability sampling methods (e.g., online advertisements, snowball sampling), which are unlikely to gain an accurate picture of the young adult population. Thus, to better understand the pandemic’s impact on the mental well-being of young adults from ethnic minority and majority groups, longitudinal research is needed that utilises information from multiple pre-pandemic assessments on a large and representative sample.

### The present study

Drawing on a large representative sample of young adults in Germany, this study asks to what extent young adults’ mental well-being had changed at the early stage of the pandemic and what role pandemic-related stressors may have played in any declines. Following participants from late adolescence to young adulthood, we draw on three assessment points to examine ethnic group differences in changes in the years before the pandemic and at the early stage of the pandemic. Our aims were threefold. First, we aimed to clarify if the mental well-being of minority groups fared worse than the majority during the early stage of the pandemic. Second, we aimed to identify whether pandemic-related stressors were related to declines in mental well-being. Third, we aimed to identify if minority groups were more exposed to stressors than the majority and whether these differences could explain any links between minority status and poorer mental well-being.

### The early COVID-19 situation in Germany

The first cases of COVID-19 were detected in Germany at the end of January 2020, followed by a substantial growth in cases by mid-March 2020 [35]. To inhibit the spread of infection, the German government implemented mitigation measures such as the closure of educational and cultural institutions and facilities on March 16 [6, 31]. The following week, further restrictions were imposed, including prohibiting gatherings of more than two people from different households, and closing all nonessential businesses and facilities [31]. Although the nationwide lockdown ended on April 19 [19], many restrictions remained, especially in regions with high infection rates [7]. Data collection for the current study commenced on April 22, thus, directly after many respondents had completed the severest lockdown period but while the lives of young adults were still heavily constrained by restrictions, such as online classes for tertiary students, working from home requirements, and limited social gatherings.

## Methods

### Participants and procedure

**Fig. 1:**
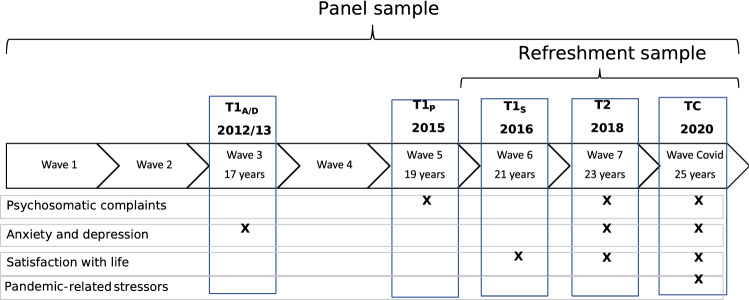
CILS4EU waves used in the current study and corresponding time points; A/D: Anxiety/Depression; P: Psychosomatic complaints; S: Life satisfaction; Approximate age in years presented

We draw on German data from the Children of Immigrants Longitudinal Survey in Four European Countries [11, 23, 24]. CILS4EU is a long-term project addressing young people’s structural and social integration that started when respondents were aged 14–16 years in 2010. To enable the study of youth with diverse backgrounds, children of immigrants were oversampled using a stratified probability cluster design (see [11, 25]). Participants in the present study include respondents from the original sample as well as a refreshment sample that was added in 2016 in the German follow-up study CILS4EU-DE (see [25]). The sample is nationally representative when using survey weights designed to adjust for different selection probabilities. We utilize the CILS4COVID survey data that was collected mainly during April–May 2020, when respondents were aged 24–26 years (*M*_*age*_ = 25). This survey inquired about respondents’ social, economic and health situation during the onset of the pandemic. Of the 5254 respondents invited to participate via web or post, 3517 participated in CILS4COVID (67% response rate; [43]). For this study, their responses were matched to responses on mental well-being in pre-pandemic surveys: 2018 (*M*_*age*_ = 23, wave 7), 2016 (*M*_*age*_ = 21, wave 6), 2015 (*M*_*age*_ = 19, wave 5) and 2012 (*M*_*age*_ = 17, wave 3). Pre-pandemic responses for the refreshment sample are only available for 2018 and 2016. See Fig. 1 for an overview of the waves used in this study. Survey data are available at the GESIS data archive (www.gesis.org; ZA5353 data file).

### Sample description

The CILS4COVID sample (59% female; 54% refreshment sample) included 54.2% native Germans, 7.7% first generation immigrants, 26.7% second generation immigrants, and 10.5% third generation immigrants (0.9% could not be classified). Most respondents (72.8%) had obtained an upper secondary degree, while 20.6% had an intermediate and 4.2% a lower secondary degree (2.4% missing). At the onset of the pandemic (TC), 42.3% were employed full-time or part-time, 38.9% were studying, 8.2% had an apprenticeship or work-related training, 4.6% were unemployed or inactive, and 3.9% did something else (2% missing).

### Measures

#### Minority groups

The majority and minority groups were derived from respondents’, parents’ and grandparents’ country of birth [16]. Participants were assigned to the German majority if neither they nor their parents or grandparents were born outside Germany (54.2%). Remaining participants were assigned to one of four categories: Former Soviet Union and Central and Eastern Europe (FSU/CEE, 18.4%); Turkey, the Middle East and Africa (Turkey/ME/Africa, 14.4%); Other European countries and the Americas (Other European/Americas, 9.3%); and Asia (2.3%). No information was available for 1.5% of the sample. These categories were chosen to reflect potential heterogeneity among minority groups regarding pandemic-related stressors, socioeconomic positioning and migration reasons, while maintaining sufficiently large cell sizes. See Additional file 1 for detailed information about countries of origin in each category.

#### Mental well-being outcomes

We used all indicators of mental well-being for which three waves of measurement were available, including the wave during the early stage of the pandemic (TC) and the two most recent pre-pandemic waves (T1_pre-pandemic_ and T2_pre-pandemic_). This included psychosomatic complaints, anxiety, depression and life satisfaction. As the inclusion of the mental well-being indicators differed between earlier waves, the wave utilised as T1 differs between outcomes (see Fig. 1).

*Psychosomatic complaints.* Psychosomatic complaints were assessed in 2015 (T1_P_), 2018 (T2) and 2020 (TC). Participants indicated how often they experienced headaches, stomach aches and difficulties falling asleep “… in the last 6 months” [T1_P_, T2] or “…in the last 2 months” [TC]. The response options were *Never, Less often, Once or several times a month, Once or several times a week* or *Every day*. We computed mean scores (α_T1P_ = 0.70, α_T2_ = 0.63, α_TC_ = 0.64), with higher values indicating more frequent psychosomatic complaints.

*Anxiety and depression.* Anxiety and depression were assessed in 2012 (T1_A/D_), 2018 (T2) and 2020 (TC). Anxiety was measured with two items: “I feel very worried” and “I feel anxious” (*r*_T1A_ = 0.36, *r*_T2_ = 0.44, *r*_TC_ = 0.56, *p*s < 0.001), and depression was measured with two items: “I feel depressed” and “I feel worthless” (*r*_T1D_ = 0.49, *r*_T2_ = 0.53, *r*_TC_ = 0.51, *p*s < 0.001). Response options ranged from 1 (*often true*) to 4 (*never true*). Mean scores were computed, with higher values indicating greater anxiety and depression.

*Satisfaction with life.* Life satisfaction was assessed in 2016 (T1_S_), 2018 (T2) and 2020 (TC) with the single question “On a scale from 1 to 10 how satisfied are you with your life in general?”. Response options ranged from 1 (*very unsatisfied*) to 10 (*very satisfied*).

#### Pandemic-related stressors

At TC respondents completed items assessing four pandemic-related stressors: personal financial and health worries, perceived changes in discrimination, and contact with COVID-19.

*Financial and health worries.* Respondents were asked: “Are you more or less worried about the following things since the beginning of the Corona pandemic?: Your financial situation” and “your health”. Response options ranged from 1 (*far more worried*) to 5 (*far less worried*). Responses were recoded so that higher values indicated increases in personal worries.

*Perceived ethnic discrimination.* Respondents were asked: “Since the beginning of the Corona pandemic, do you feel you have been treated unfairly or discriminated against because of your ethnicity more often than before?”. The response categories were: *Yes*, *Somewhat yes*, *Somewhat no*, *No* and *I am never treated unfairly or discriminated against because of my ethnicity*. A three-category variable was generated representing *never discriminated against* (0), *no change in discrimination* (1) and *increase in discrimination* (2), which was subsequently dummy coded.

*Contact with COVID-19.* Respondents were asked “Have you or someone you know been diagnosed with the coronavirus?”. Multiple response alternatives were available, including: *Yes, myself*; *Yes, someone from my family*; *Yes, someone from my circle of friends*; *Yes, someone from my circle of acquaintances*; *No*; *I do not want to say*. Less than 1% (0.4%) had tested positive for COVID-19, 3.3% had a positive case in the family, 6.1% had a friend who tested positive, and 16.4% knew an acquaintance who tested positive. Responses from the first four categories were summed, so that scores ranged from 0 to 4, with higher values indicating greater contact with COVID-19.

#### Control variables

The following variables were used to control for sociodemographic factors: respondents’ gender, age (year of birth, mean centred), secondary educational degree (lower, intermediate, upper), and parental occupational status (highest ISEI score of parents, mean centred).

### Analytical strategy

Individual fixed effects regressions were performed for each outcome to identify changes in well-being prior to the pandemic (i.e., from T1 to T2) and changes at the early stage of the pandemic (i.e., from T2 to TC). Fixed effects regressions bring the advantage of controlling for time-invariant confounders. To examine if changes differed between minority groups and the German majority, interactions between time and minority group were included. Dummies indicated each time point, each minority group, and interactions between the time points and minority groups. T2 and German majority were the reference categories. If the pandemic had a worse effect on minorities, we would expect group differences reflecting a disadvantage for minorities to be most notable in changes at the pandemic assessment (from T2 to TC). Although it is plausible that changes prior to the pandemic (from T1 to T2) were similar across groups, it is also conceivable that these changes were already worse for minorities, due to factors such as discrimination and other challenges experienced during transitions to tertiary studies and employment. In either scenario, if group differences in changes arose or accelerated at the pandemic assessment (between T2 and TC), this would signal a more detrimental impact of the pandemic on minorities than the ethnic German majority. The fixed effects regression models applied sampling weights and were performed in R using the package ‘plm’ [12]. These analyses excluded respondents with missing information on ethnic background (*n* = 52) and survey weights (*n* = 37), as well as those missing information on the mental well-being outcomes at two or three time points. This resulted in the following analytical samples: psychosomatic complaints *n* = 3185, anxiety *n* = 3179, depression *n* = 3169 and life satisfaction *n* = 3198. Psychosomatic complaints, anxiety and depression utilise the panel sample for changes between T1 to T2 but the panel and refreshment samples (i.e., full analytical samples) for changes between T2 and TC.

We then examined the role of pandemic-related stressors in changes in mental well-being at the early stage of the pandemic. To identify if pandemic-related stressors were more common among minorities and whether such differences might explain any links between minority status and declines in well-being, we tested a mediation model using path analysis. Given that the pandemic-related stressors were measured only at TC, we examined mental well-being difference scores based on responses from T2 and TC, with higher values indicating an increase in the well-being outcome. The mediation analysis was conducted in Mplus 7.4 [32] using the MLR estimator and MODEL INDIRECT command. All outcomes and mediators were examined within a single model, including weights, and controlling for respondents’ gender, age, secondary education, and parental occupational status. Cases with missing data on weights, exogenous variables and all well-being outcomes were deleted listwise, and full information maximum likelihood estimation (FIML) was used to handle remaining missing data on the dependent variables, resulting in an analytical sample of *n* = 3393.

## Results

### Descriptive statistics

Descriptives for the well-being outcomes at each time point and the pandemic-related stressors are shown in Table 1 by the whole sample and by ethnic group. Correlations among the main variables are reported in Additional file 2. Prior to Covid (T1, T2), mean rates of psychosomatic complaints were higher among respondents with origins in Turkey/ME/Africa than the German majority. Furthermore, at T1 each minority group had significantly lower life satisfaction than the German majority. At the pandemic assessment (TC), there were no significant group differences in mean rates of psychosomatic complaints or depression. However, anxiety was significantly higher among Turkey/ME/Africa minorities than the German majority. Although life satisfaction was relatively high across all groups, the mean for Turkey/ME/Africa minorities was lower than that of the German majority.

**Table 1:** Means and standard deviations of the mental well-being indicators and pandemic-related stressors, *M*(*SD*) (weighted)

	Total	German	FSU/CEE	Other European/Americas	Asia	Turkey /ME Africa
Psychosomatic complaints						
T1_P_ (M_age_ = 17)	2.70 (0.83)	2.62 (0.85)	2.78 (0.77)	2.88 (0.67)^a^	2.17 (0.71)	2.92 (0.84)^a^
T2 (M_age_ = 23)	2.58 (0.79)	2.55 (0.78)	2.63 (0.76)	2.66 (0.83)	2.33 (0.75)	2.69 (0.85)^a^
TC (M_age_ = 25)	2.48 (0.85)	2.48 (0.84)	2.48 (0.83)	2.48 (0.90)	2.49 (0.78)	2.52 (0.91)
Anxiety						
T1_A_ (M_age_ = 19)	2.41 (0.70)	2.41 (0.71)	2.43 (0.64)	2.44 (0.74)	2.30 (0.80)	2.37 (0.73)
T2 (M_age_ = 23)	2.60 (0.69)	2.58 (0.68)	2.66 (0.73)	2.68 (0.70)	2.37 (0.71)	2.62 (0.68)
TC (M_age_ = 25)	2.21 (0.75)	2.19 (0.73)	2.22 (0.79)	2.23 (0.73)	2.16 (0.65)	2.36 (0.79)^a^
Depression						
T1_D_ M_age_ = 19)	1.90 (0.72)	1.91 (0.70)	1.86 (0.72)	1.99 (0.71)	1.62 (0.66)	1.88 (0.82)
T2 (M_age_ = 23)	2.04 (0.72)	2.04 (0.69)	2.01 (0.74)	2.11 (0.77)	1.84 (0.74)	2.09 (0.81)
TC (M_age_ = 25)	1.79 (0.79)	1.78 (0.78)	1.75 (0.75)	1.88 (0.83)	1.71 (0.70)	1.88 (0.86)
Life satisfaction						
T1_S_ (M_age_ = 21)	7.78 (1.57)	7.90 (1.45)	7.68 (1.59)^a^	7.54 (1.80)^a^	7.26 (1.60)^a^	7.39 (2.01)^a^
T2 (M_age_ = 23)	7.26 (2.06)	7.32 (2.05)	7.30 (1.96)	6.96 (2.27)	7.28 (1.75)	6.94 (2.15)
TC (M_age_ = 25)	7.19 (2.13)	7.26 (2.06)	7.21 (2.06)	7.04 (2.43)	7.20 (1.74)	6.69 (2.48) ^a^
Financial worries	3.39 (1.00)	3.34 (0.96)	3.43 (1.02)	3.54 (1.03)	3.39 (1.01)	3.53 (1.24)
Health worries	3.44 (0.87)	3.41 (0.79)	3.42 (0.98)	3.45 (0.85)	3.75 (0.84)	3.74 (1.13)^a^
Increase in discrimination	0.03 (0.18)	0.02 (0.15)	0.02 (0.14)	0.02 (0.12)	0.40 (0.49)^a^	0.09 (0.29)^a^
No change in discrimination	0.44 (0.50)	0.39 (0.49)	0.47 (0.50)^a^	0.48 (0.50)	0.37 (0.49)	0.72 (0.45)^a^
Contact with Covid-19	0.27 (0.52)	0.30 (0.54)	0.21 (0.49)^a^	0.26 (0.54)	0.21 (0.42)	0.16 (0.39)^a^

^a^Significantly different to the German majority (*p* < .003) based on two-tailed t-tests with a Bonferroni adjustment to reduce the likelihood of type I error when making multiple comparisons; Range-Psychosomatic complaints = 1–5, anxiety and depression = 1–4; life satisfaction = 1–10; financial and health-related worries = 1–5; Discrimination = 0–1; Contact with COVID-19 = 0–4

When considering all three waves together, several trends in mental well-being are observed. The means for anxiety and depression appear to increase between T1 and T2 but decrease at TC among all groups. In contrast, mean rates of psychosomatic complaints seem to decrease across time among all groups except among Asian minorities, whose mean scores increased. Life satisfaction appeared to have a negative gradient across groups, with mean rates generally decreasing over time. However, as these trends are purely descriptive and do not control for confounding factors, we examine changes in mental well-being in more detail in the following section.

### Group differences in changes in mental well-being before and at the early stage of the pandemic

**Fig. 2:**
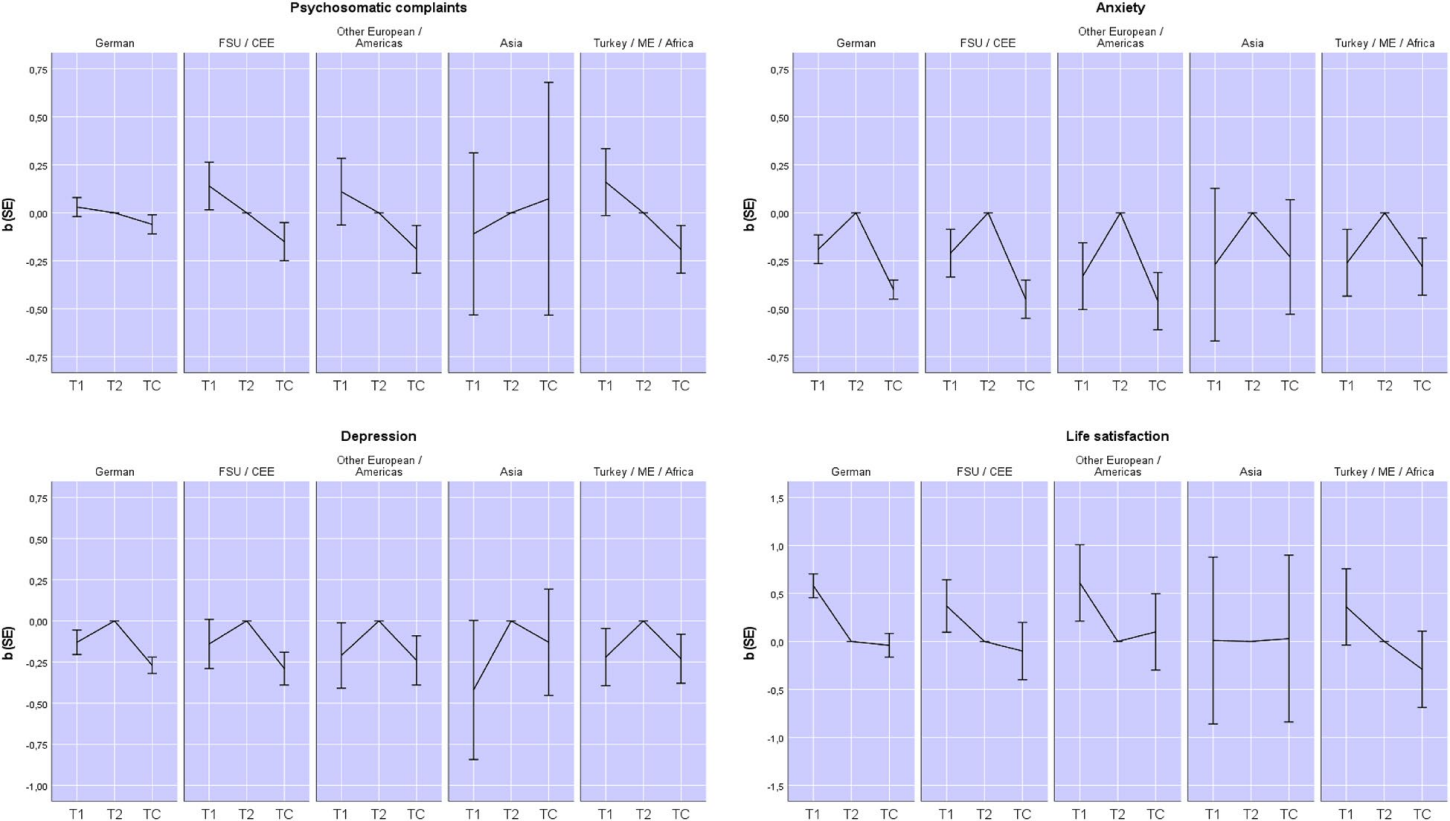
Unstandardized effects (*b*) and standard errors (*SE*) for psychosomatic complaints, anxiety, depression and life satisfaction across groups and time

Results from the individual fixed effects regressions testing group differences in changes in mental well-being are shown in Table 2 and illustrated in Fig. 2. The T1 estimates indicate changes in well-being prior to the pandemic (T1 to T2) across all groups. Since T2 was the reference category, positive values indicate a decrease and negative values an increase in the respective outcome. Between the two pre-pandemic assessments, changes in psychosomatic complaints were not significant, yet anxiety and depression increased, while life satisfaction decreased significantly. The T1 × minority group interactions indicate that psychosomatic complaints decreased more strongly among FSU/CEE respondents than the German majority. The coefficients for T1 × Asia suggest that within-person changes in psychosomatic complaints and depression prior to the pandemic were worse for Asian respondents than the German majority, although these differences did not reach statistical significance.

**Table 2:** Changes in mental well-being prior to the pandemic and at the early stage of the pandemic (weighted)

	Psychosomatic complaints	Anxiety	Depression	Life satisfaction
T1 (prior to the pandemic)	0.03 (0.02)	− 0.19 (0.03)***	− 0.13 (0.03)***	0.58 (0.05)***
TC (early stage of pandemic)	− 0.06 (0.02)***	− 0.40 (0.02)***	− 0.27 (0.02)***	− 0.04 (0.05)
T1 × FSU/CEE	0.11 (0.05)*	− 0.02 (0.05)	− 0.01 (0.06)	− 0.21 (0.11)
T1 × Other European/Americas	0.08 (0.07)	− 0.14 (0.07)	− 0.08 (0.08)	0.03 (0.16)
T1 × Asia	− 0.14 (0.17)	− 0.08 (0.16)	− 0.29 (0.17)	− 0.57 (0.35)
T1 × Turkey/ME/Africa	0.13 (0.07)	− 0.07 (0.07)	− 0.09 (0.07)	− 0.22 (0.16)
TC × FSU/CEE	− 0.09 (0.04)*	− 0.05 (0.04)	− 0.02 (0.04)	− 0.06 (0.12)
TC × Other European/Americas	− 0.13 (0.05)*	− 0.06 (0.06)	0.03 (0.06)	0.14 (0.16)
TC × Asia	0.21 (0.12)	0.17 (0.12)	0.14 (0.13)	0.07 (0.35)
TC × Turkey/ME/Africa	− 0.13 (0.05)*	0.12 (0.06)*	0.04 (0.06)	− 0.25 (0.16)

Unbalanced panel	n = 3185 T = 2–3 N = 7766	n = 3179 T = 2–3 N = 7746	n = 3169 T = 2–3 N = 7720	n = 3198 T = 2–3 N = 9559

	*F*(10,4571) = 12.21***	*F*(10,4557) = 77.63***	*F*(10,4541) = 30.23***	*F*(10,6351) = 25.08***
	*R*^*2*^ = .023	*R*^*2*^ = .139	*R*^*2*^ = .042	*R*^*2*^ = .044

Table shows unstandardized coefficients and standard errors in parentheses; T2 and German are reference categories; n = the number of respondents who responded at least twice; T: how often an individual was observed; N: the total number of observations in the pooled model (across time); *p < .05, **p < .01, ***p < .001

The TC estimates indicate changes between the most recent pre-pandemic and the early pandemic assessments (T2 to TC). Positive values represent an increase, while negative values represent a decrease in the respective outcome. Overall, psychosomatic complaints, anxiety, and depression had decreased at the early stage of the pandemic compared to the most recent pre-pandemic assessment, while life satisfaction had not significantly changed. The TC × ethnic minority interactions show that psychosomatic complaints decreased more strongly among FSU/CEE, other European/Americas and Turkey/ME/Africa minorities than the German majority. Anxiety however, decreased less strongly among Turkey/ME/Africa minorities than for the German majority. The coefficients for TC × Asia suggest that young adults of Asian descent had increased psychosomatic complaints and weaker decreases in anxiety and depression. Although these group differences were not statistically significant, we emphasise them because the pattern is indicative of meaningful variation, and the relatively large size of the coefficients and standard errors suggest that the lack of statistical significance may be due to the small size of the Asian sample.

In summary, changes in mental well-being prior to the pandemic were mostly similar between the German majority and minority groups. With the exception of psychosomatic complaints, anxiety and depression for Asian respondents and anxiety for the Turkey/ME/Africa minorities, changes at the early stage of the pandemic were not worse for minorities.

### Pandemic-related stressors and changes in mental well-being

To examine whether pandemic-related stressors were related to changes in mental well-being at the early stage of the pandemic and could explain links between minority group membership and poorer mental well-being, a path analysis was performed using mental well-being change scores from T2 to TC. This model, which included correlations among the well-being outcomes and correlations among the pandemic-related stressors, showed excellent fit (χ^2^(*df*) = 9.06(7), *p* = 0.248, RMSEA = 0.009, 90% CI [0.000, 0.024], CFI = 0.998, TLI = 0.958, SRMR = 0.005). The direct path coefficients are shown in Table 3.

**Table 3:** Direct pathways among minority groups, pandemic-related stressors and changes in mental well-being (weighted)

	Financial worries	Health worries	Increase in discrimination	No change in discrimination	Contact with COVID-19
Minority group → stressors					
FSU/CEE	0.06 (0.08)	0.00 (0.07)	− 0.00 (0.01)	0.07 (0.04)	− 0.08 (0.04)^*^
Other European/Americas	0.19 (0.09)^*^	0.01 (0.08)	− 0.01 (0.01)	0.09 (0.05)^*^	− 0.03 (0.05)
Asia	0.09 (0.20)	0.40 (0.14)^**^	0.37 (0.09)^***^	− 0.02 (0.09)	− 0.07 (0.06)
Turkey/ME/Africa	0.13 (0.10)	0.27 (0.09)^**^	0.07 (0.02)^**^	0.29 (0.04)^***^	− 0.11 (0.04)^**^
	*R*^*2*^ = .02 ^*^	*R*^*2*^ = .02^*^	*R*^*2*^ = .08^**^	*R*^*2*^ = .05^***^	*R*^*2*^ = .02^***^

	Psychosomatic complaints	Anxiety	Depression	Life satisfaction	
Stressors → well-being					
Financial worries	0.01 (0.02)	0.10 (0.02)^***^	0.08 (0.03)^**^	− 0.17 (0.07)^*^	
Health worries	0.01 (0.03)	0.21 (0.02)^***^	0.12 (0.03)^***^	− 0.06 (0.08)	
Increase in discrimination	0.10 (0.15)	0.17 (0.15)	0.36 (0.14)^**^	0.13 (0.39)	
No change in discrimination	0.02 (0.04)	− 0.01 (0.04)	0.03 (0.05)	− 0.14 (0.15)	
Contact with Covid-19	0.12 (0.06)^*^	0.11 (0.04)^**^	0.09 (0.04)^*^	0.12 (0.13)	
Minority group → well-being					
FSU/CEE	− 0.06 (0.06)	− 0.05 (0.06)	− 0.02 (0.06)	− 0.03 (0.18)	
Other European/Americas	− 0.11 (0.07)	− 0.10 (0.07)	0.02 (0.08)	0.14 (0.26)	
Asia	0.23 (0.15)	0.02 (0.12)	− 0.07 (0.13)	− 0.07 (0.28)	
Turkey/ME/Africa	− 0.07 (0.08)	0.07 (0.07)	0.00 (0.09)	− 0.31 (0.25)	
	*R*^*2*^ = .03 ^**^	*R*^*2*^ = .09^***^	*R*^*2*^ = .04^***^	*R*^*2*^ = .02^*^	

Table shows unstandardized coefficients and standard errors in parentheses; Minority group—The German majority is the reference category; All models control for gender, age, participants’ education, and parental occupational status; * p < .05, **p < .01, ***p < .001

We found that the Asia, and to a lesser degree, Turkey/ME/Africa, minorities perceived a significantly greater increase in ethnic discrimination than the German majority. Furthermore, young adults with origins in Turkey/ME/Africa, and to a lesser degree Other Europe/Americas, were more likely than the German majority to report no change in discrimination, indicative of an ongoing level of discrimination among these groups. Respondents with origins in Asia and Turkey/ME/Africa reported a greater increase in health worries than the German majority, while those with origins in Other Europe/Americas reported a greater increase in financial worries. Finally, the FSU/CEE and Turkey/ME/Africa minorities reported less contact with COVID-19 than the German majority.

Turning to associations between the pandemic-related stressors and mental well-being, financial worries were associated with increases in anxiety and depression, as well as decreases in life satisfaction. Health worries were associated with increases in anxiety and depression. Increased ethnic discrimination was linked to an increase in depression, while greater contact with COVID-19 was associated with increases in psychosomatic complaints, anxiety and depression.

Although no significant direct effects between minority group membership and mental well-being were identified in the path analysis, indirect effects were found via pandemic-related stressors. As shown in Table 4, among Asian minorities, significant effects were found on anxiety via health worries, and on depression via health worries and discrimination. Furthermore, for respondents with origins in Turkey/ME/Africa, effects on anxiety and depression were found via health worries. Neither direct nor indirect effects from the FSU/CEE and Other European/Americas minorities on mental well-being were found. See Additional file 3 for full mediation results.

**Table 4:** Total, direct and indirect effects of minority groups on changes in mental well-being via pandemic-related stressors

	Anxiety	Depression
Asia		
Total effect	0.17 (0.09)	0.11 (0.13)
Direct effect	0.02 (0.12)	− 0.07 (0.13)
Total indirect	0.15 (0.07)*	0.18 (0.07)*
Specific indirect effects via		
Financial worries	0.01 (0.02)	0.01 (0.02)
Health worries	0.08 (0.03)**	0.05 (0.02)*
Increase in discrimination	0.06 (0.06)	0.13 (0.06)*
No change in discrimination	0.00 (0.00)	− 0.00 (0.00)
Contact with COVID-19	− 0.01 (0.01)	− 0.01 (0.01)
Turkey/ME/Africa		
Total effect	0.14 (0.07)*	0.07 (0.09)
Direct effect	0.07 (0.07)	0.00 (0.09)
Total indirect	0.07 (0.03)*	0.07 (0.03)*
Specific indirect effects via		
Financial worries	0.01 (0.01)	0.01 (0.01)
Health worries	0.06 (0.02)**	0.03 (0.01)*
Increase in discrimination	0.01 (0.01)	0.02 (0.01)
No change in discrimination	− 0.00 (0.01)	0.01 (0.01)
Contact with COVID-19	− 0.01 (0.01)	− 0.01 (0.01)

Table shows unstandardized effects and standard errors in parentheses

* *p* < .05, ** *p* < .01, *** *p* < .001

### Robustness checks

To better exploit the large CILS4COVID sample, we used the panel and refreshment samples to examine changes in psychosomatic complaints, depression, and anxiety from T1 to TC, but only the panel sample for changes between T1 to T2. The robustness of our findings were tested by performing sensitivity analyses that only used respondents with information available at all three time-points (i.e., excluding the refreshment sample). These found that the TC and Turkey/ME/Africa interaction on anxiety reduced and was no longer significant, but otherwise came to substantively similar findings (see Additional file 4). Furthermore, as changes in well-being may be confounded by cultural differences in family building, the fixed effects regressions were also re-run controlling for having a child or marrying. However, these analyses did not change the current findings (see Additional file 5).

The path analysis used FIML to handle missing data on dependent variables, allowing us to maximise the analytical sample size by retaining participants with partially missing data. To ensure that anomalies had not arisen during the estimation process, a path analysis was performed using only participants with complete information on all mental well-being outcomes (*n* = 3100). This produced results that were nearly identical to the presented findings (see Additional file 6).

## Discussion

This study examined the impact of the COVID-19 pandemic on young adults’ mental well-being and explored potential disparities between ethnic minority and majority groups. Drawing on a large representative sample of young adults in Germany, we asked whether minorities fared worse than the majority during the early stage of the pandemic. Furthermore, we investigated whether minorities experienced greater exposure to pandemic-related stressors, and if greater exposure made young adults from the minority population more vulnerable to declines in mental well-being than young adults from the majority population.

Using three waves of data, we found that overall, young adults’ mental well-being had improved at the early stage of the pandemic compared to 2 years earlier. Although life satisfaction remained stable, psychosomatic complaints, anxiety and depression had decreased. Furthermore, these improvements in mental well-being at the pandemic assessment followed a trend of worsening anxiety, depression, and life satisfaction prior to the pandemic. These findings challenge previous reports of greater mental health problems [4, 20, 30] and declines in mental well-being among young adults (e.g., [13, 17, 37]) early in the pandemic. By using longitudinal data with two pre-pandemic waves from a representative sample of young adults, we were able to illustrate that despite the disruptions brought about by the pandemic, our sample of ~ 25-year-olds experienced worse mental well-being (on average) before the onset of the pandemic. Such patterns might be masked in studies using nonrepresentative samples, cross-sectional designs or longitudinal designs with only one pre-pandemic wave.

Our findings also revealed few ethnic group differences in changes in mental well-being among young adults prior to and during the pandemic, which is consistent with longitudinal studies of older adults (e.g., [5, 45]). Although some disadvantages were found for young adults with origins in Asia, Turkey, the Middle East and Africa, there was no evidence of stronger declines in mental well-being among ethnic minorities at the early stage of the pandemic. Instead, there was evidence of weaker improvements in some aspects of mental well-being among these ethnic minority groups. A cross-sectional analysis of our data might have concluded that Turkish, Middle Eastern and African minorities had worse anxiety and life satisfaction than the German majority at the early stage of the pandemic. However, our methodological approach allowed us to clarify that anxiety among these minorities had improved (on average) between pre-pandemic and pandemic assessments, albeit to a lesser extent than for the German majority, and that a lower rate of life satisfaction among Turkish, Middle Eastern and African minorities already existed before the pandemic.

While the findings suggest that changes in psychosomatic complaints and depression at the pandemic assessment were less positive for respondents of Asian descent compared to the German majority, similar disadvantages were also observed in changes prior to the pandemic. Thus, by examining two waves of pre-pandemic data, we can infer that these disparities in changes at the early stage of the pandemic are unlikely to have resulted solely from the pandemic. It is noteworthy that the mental well-being, particularly psychosomatic complaints, of Asian respondents seemed to follow a less positive trajectory from adolescence than other groups. Although the estimates for the Asian minorities did not reach statistical significance, this was likely due to power limitations from the small size of this group. As this pattern deserves further attention, future research should test if these patterns replicate with a larger sample of Asian minorities. If disadvantages are found, perhaps group differences can be explained by variation in cultural norms or pressures regarding life transitions during young adulthood, such as living arrangements, completing higher education or entering the labour market.

Despite finding that young adults generally had better mental well-being at the early stage of the pandemic than two years prior, respondents with greater exposure to pandemic-related stressors were less likely to experience positive changes in well-being. Overall, young adults who reported greater contact with COVID-19, financial worries and health worries experienced increased anxiety and depression. Furthermore, increased discrimination and financial worries were associated with increased depression and decreased life satisfaction, respectively. The current findings thus support earlier cross-sectional studies indicating that worries, discrimination and contact with COVID-19 are related to poorer mental well-being during the pandemic (Cao et al., 2020; [28, 29, 40]). We also extend previous cross-sectional findings by identifying how each stressor related to declines in specific aspects of mental well-being. Anxiety and depressive symptoms, for example, were associated with a larger number of pandemic-related stressors than psychosomatic complaints and life satisfaction. The latter two aspects of mental well-being may be more resilient to transient or new challenges than anxiety and depression. Data that tracks young adults across the pandemic’s duration could clarify if increased health worries or discrimination that are sustained over a longer period negatively impact psychosomatic complaints or life satisfaction.

We found that overall, minority groups had greater exposure to pandemic-related stressors than the majority population. However, exposure varied between the minority groups and the results indicate that certain minorities in Germany are more vulnerable than others. Respondents with origins in Asia, Turkey, the Middle East and Africa reported a greater increase in discrimination and health worries, while those with backgrounds in other European countries and the Americas reported greater financial worries. Moreover, the mediation analysis showed that an increase in health worries among young adults with origins in Asia, Turkey, the Middle East and Africa contributed to increases in anxiety and depression among these groups at the pandemic assessment. Furthermore, a greater increase in discrimination among young adults of Asian descent also contributed to increases in depression among this group. Thus, although we identified few ethnic group differences in how mental well-being changed overall, greater exposure to pandemic-related stressors rendered Asian, Turkish, Middle Eastern and African minorities more vulnerable to less positive changes in mental well-being at the early stage of the pandemic.

Compared to young adults with origins in Europe, the Americas, or the former Soviet Union, those from Asia, Turkey, the Middle East and Africa experienced more ethnic discrimination. The high probability of Asian minorities to have experienced increased discrimination at the early stage of the pandemic was striking, as was the considerably high likelihood of ongoing discrimination among Turkish, Middle Eastern and African minorities. These findings demonstrate the longstanding problem of racism in society that these young adults are subjected to, and that the pandemic brought about amplified experiences of discrimination among Asian minorities. Although a large proportion from the ethnic majority reported some level of discrimination, this is not a new finding and is consistent with previous research [2, 33].

The unexpected finding of less contact with COVID-19 among the former Soviet Union, Central and Eastern Europe and Turkish, Middle Eastern and African minorities needs further investigation. If these groups had poorer access to healthcare services that provided testing or contact tracing than the German majority, perhaps their awareness of contact with COVID-19 may have been lower, despite rates of infection being higher among disadvantaged minority groups. However, the elevated health worries among Turkish, Middle Eastern and African minorities might be attributed to news and social media coverage describing higher infection rates among disadvantaged ethnic/racial and immigrant groups. As Asian minorities in Germany are not typically socioeconomically disadvantaged, we speculate that their higher health worries stem from closer psychological or social connections to the world region where COVID-19 or possibly even the previous SARS outbreak began.

Key strengths of the current study include the large and nationally representative sample, and the use of longitudinal survey data covering the early stage of the pandemic and two pre-pandemic assessments. These advantages permitted analyses of within-person changes to provide a more accurate indication than is otherwise currently available on how ethnic minority and majority young adults’ mental well-being may have been impacted at the early stage of the pandemic. In addition, the consideration of multiple pandemic-related stressors and aspects of mental well-being enabled a comprehensive examination of potential disparities between ethnic minorities and the majority.

Despite these strengths, the following limitations must be acknowledged. First, as information on respondents’ mental well-being directly before the pandemic was not available, we cannot draw conclusions about more immediate changes. Given that our most recent pre-pandemic assessment was approximately 2 years prior to the pandemic, the exact timing for changes in well-being is unclear. Studies using a shorter interval may find that young adults’ mental well-being did decrease after the onset of the pandemic. Nevertheless, the current findings present a unique developmental picture by showing how mental well-being had changed at the early stage of the pandemic, in the context of changes from adolescence and the years leading up to the pandemic.

In addition, mental well-being is often considered to be vulnerable as young adults attempt to negotiate various developmental milestones, such as self-identity achievement, completing education, gaining employment, moving out of home, and forming more serious romantic partnerships [34]. As the mitigation measures may have temporarily delayed these transitions, future studies could examine if some young adults’ mental well-being improved due to (initially) reduced pressures to perform socially, academically, or career-wise. A notable degree of heterogeneity in changes existed (results not shown), thus individual differences in reactions to the pandemic are worth following up to further understanding of risk and resilience among young adults.

A second limitation is that our data do not allow us to track changes in mental well-being within the pandemic. The continuation and re-introduction of mitigation measures in Germany throughout the pandemic’s duration may have had detrimental effects on young adults’ mental well-being, particularly minorities’. Data with multiple pandemic waves is needed to test this possibility. Thirdly, we did not focus on pre-existing mental health problems, although these are a risk factor for declines in mental well-being during the pandemic (e.g., [26, 37]). Some young adults with mental health problems at earlier waves may have received treatment or been in contact with healthcare providers. To provide a deeper understanding of the pandemic’s impact on inequalities in mental well-being, future studies could examine changes in well-being specifically among ethnic minority and majority young adults with pre-existing mental health problems and what role health services played in supporting them during the pandemic. Finally, as this study is based on a German sample, generalisations to young adults in other countries may be limited. Cross-country comparisons are needed to examine what role country specific mitigation strategies in the context of different cultural, economic, technological, and social welfare factors (e.g., healthcare access, unemployment benefits) played in young adults’ initial experiences during the pandemic.

## Conclusions

Despite the challenges presented by the COVID-19 pandemic, overall, we find a picture of resilience among young people at the early stage of the pandemic. The mental well-being of young adults from ethnic minority and majority groups had generally improved compared to pre-pandemic assessments. Nevertheless, in certain aspects, young adults with a Turkish, Middle Eastern, African or Asian background fared less well than the German majority. The findings point to a need to address the ethnic discrimination these minority groups face and the increased health worries that contributed to anxiety and depression for some young adults in these groups. It would be beneficial to develop strategies to help young adults who have experienced increased discrimination, as well as health and financial worries, to transition out of the pandemic positively. As the pandemic continued, pandemic-related stressors may have intensified, and mental well-being may have deteriorated. Thus, further longitudinal research is required to understand the continuing impact of the pandemic on mental well-being among ethnic minority young adults.

## Supplementary Information

**Additional file 1.** Countries of origin within each minority category.

**Additional file 2.** Correlations among main variables.

**Additional file 3.** Total, direct and indirect effects of minority groups on changes in well-being via pandemic-related stressors.

**Additional file 4.** Robustness checks for the fixed effects regressions based on respondents with no missing information.

**Additional file 5.** Robustness checks for the fixed effects regressions including changes in marriage and parental status.

**Additional file 6.** Robustness check for the path analysis using only respondents with complete data on all mental well-being outcomes.

## Data Availability

The datasets generated and/or analysed during the current study are available in the GESIS data archive, www.gesis.org; ZA5353 data file.
