# Supplementary material for: Changes in young adults' mental well-being before and during the early stage of the COVID-19 pandemic: disparities between ethnic groups in Germany
Source: Child Adolesc Psychiatry Ment Health. 2021 Nov 23;15:69. doi: 10.1186/s13034-021-00418-x (PMC8609988; doi:10.1186/s13034-021-00418-x)
Supplement: Supplementary file 1 — Additional file 1. Countries of origin within each minority category. [file 13034_2021_418_MOESM1_ESM.docx]

*Additional file 1*

*Table A1.* Countries of origin within each minority category

| *Former Soviet Union and Central and Eastern Europe* | n | % |  | *Other European countries and Americas* | n | % |
| --- | --- | --- | --- | --- | --- | --- |
| Poland | 209 | 32.3 |  | Italy | 79 | 24.2 |
| Russian Federation | 120 | 18.5 |  | Austria | 37 | 11.3 |
| Serbia | 42 | 6.5 |  | Greece | 28 | 8.6 |
| Romania | 36 | 5.6 |  | Spain | 24 | 7.4 |
| Czech Republic | 30 | 4.6 |  | France | 21 | 6.4 |
| Kazakhstan | 28 | 4.3 |  | Netherlands | 18 | 5.5 |
| Ukraine | 27 | 4.2 |  | Switzerland | 15 | 4.6 |
| Czechoslovakia | 26 | 4.0 |  | United States of America | 15 | 4.6 |
| Hungary | 22 | 3.4 |  | United Kingdom of Great Britain and Northern Ireland | 14 | 4.3 |
| Bosnia and Herzegovina | 18 | 2.8 |  | Portugal | 12 | 3.7 |
| Socialist Federal Republic of Yugoslavia | 14 | 2.2 |  | Brazil | 10 | 3.1 |
| Croatia | 13 | 2.0 |  | Americas | 9 | 2.8 |
| Former German Eastern Territories | 11 | 1.7 |  | Belgium | 7 | 2.1 |
| Albania | 8 | 1.2 |  | Denmark | 5 | 1.5 |
| Bulgaria | 7 | 1.1 |  | Argentina | 3 | .9 |
| Slovakia | 5 | .8 |  | Canada | 3 | .9 |
| The Former Yugoslav Republic of Macedonia | 5 | .8 |  | Chile | 3 | .9 |
| Republic of Moldova | 4 | .6 |  | Colombia | 3 | .9 |
| Azerbaijan | 3 | .5 |  | Dominican Republic | 3 | .9 |
| USSR | 3 | .5 |  | Ireland | 3 | .9 |
| Armenia | 2 | .3 |  | Mexico | 3 | .9 |
| Belarus | 2 | .3 |  | Jamaica | 2 | .6 |
| Georgia | 2 | .3 |  | Bolivarian Republic of Venezuela | 2 | .6 |
| Kyrgyzstan | 2 | .3 |  | Australia | 1 | .3 |
| Lithuania | 2 | .3 |  | Cyprus | 1 | .3 |
| Montenegro | 2 | .3 |  | Ecuador | 1 | .3 |
| Slovenia | 2 | .3 |  | Finland | 1 | .3 |
| Estonia | 1 | .2 |  | Nicaragua | 1 | .3 |
| Latvia | 1 | .2 |  | Peru | 1 | .3 |
| Total | 647 | 100.0 |  | United States Virgin Islands | 1 | .3 |
|  |  |  |  | Total | 326 | 100.0 |
| *Turkey, the Middle East and Africa* | n | % |  | *Asia* | n | % |
| Turkey | 337 | 66.6 |  | Viet Nam | 26 | 32.5 |
| Iraq | 20 | 4.0 |  | China | 13 | 16.3 |
| Syrian Arab Republic | 17 | 3.4 |  | Sri Lanka | 9 | 11.3 |
| Islamic Republic of Iran | 16 | 3.2 |  | Thailand | 7 | 8.8 |
| Lebanon | 16 | 3.2 |  | India | 6 | 7.5 |
| Afghanistan | 14 | 2.8 |  | Republic of Korea | 5 | 6.3 |
| Morocco | 14 | 2.8 |  | Philippines | 4 | 5.0 |
| Nigeria | 7 | 1.4 |  | Bangladesh | 3 | 3.8 |
| Occupied Palestinian Territory | 6 | 1.2 |  | Indonesia | 2 | 2.5 |
| Pakistan | 6 | 1.2 |  | Nepal | 2 | 2.5 |
| Tunisia | 6 | 1.2 |  | Cambodia | 1 | 1.3 |
| Eritrea | 5 | 1.0 |  | Japan | 1 | 1.3 |
| Ghana | 5 | 1.0 |  | Lao People's Democratic Republic | 1 | 1.3 |
| Algeria | 4 | .8 |  | Total | 80 | 100.0 |
| Angola | 3 | .6 |  |  |  |  |
| Cameroon | 3 | .6 |  |  |  |  |
| Egypt | 3 | .6 |  |  |  |  |
| Africa | 2 | .4 |  |  |  |  |
| Congo | 2 | .4 |  |  |  |  |
| Democratic Republic of the Congo | 2 | .4 |  |  |  |  |
| Ethiopia | 2 | .4 |  |  |  |  |
| Gambia | 2 | .4 |  |  |  |  |
| Somalia | 2 | .4 |  |  |  |  |
| Israel | 1 | .2 |  |  |  |  |
| Jordan | 1 | .2 |  |  |  |  |
| Kenya | 1 | .2 |  |  |  |  |
| Namibia | 1 | .2 |  |  |  |  |
| Sao Tome and Principe | 1 | .2 |  |  |  |  |
| Senegal | 1 | .2 |  |  |  |  |
| Sierra Leone | 1 | .2 |  |  |  |  |
| South Africa | 1 | .2 |  |  |  |  |
| Togo | 1 | .2 |  |  |  |  |
| United Republic of Tanzania | 1 | .2 |  |  |  |  |
| Yemen | 1 | .2 |  |  |  |  |
| Kurdistan | 1 | .2 |  |  |  |  |
| Total | 506 | 100.0 |  |  |  |  |
