# Supplementary material for: Changes in young adults' mental well-being before and during the early stage of the COVID-19 pandemic: disparities between ethnic groups in Germany
Source: Child Adolesc Psychiatry Ment Health. 2021 Nov 23;15:69. doi: 10.1186/s13034-021-00418-x (PMC8609988; doi:10.1186/s13034-021-00418-x)
Supplement: Supplementary file 2 — Additional file 2. Correlations among main variables. [file 13034_2021_418_MOESM2_ESM.docx]

*Additional file 2*

*Table A2.1*. Correlations main variables: German

|  | 1 | 2 | 3 | 4 | 5 | 6 | 7 | 8 | 9 | 10 | 11 | 12 | 13 | 14 | 15 | 16 |
| --- | --- | --- | --- | --- | --- | --- | --- | --- | --- | --- | --- | --- | --- | --- | --- | --- |
| 1. Y5 PSC |  |  |  |  |  |  |  |  |  |  |  |  |  |  |  |  |
| 1. Y7 PSC | **.59** |  |  |  |  |  |  |  |  |  |  |  |  |  |  |  |
| 1. YC PSC | **.47** | **.56** |  |  |  |  |  |  |  |  |  |  |  |  |  |  |
| 1. Y3 Anxiety | **.34** | **.33** | **.25** |  |  |  |  |  |  |  |  |  |  |  |  |  |
| 1. Y7 Anxiety | **.37** | **.40** | **.30** | **.47** |  |  |  |  |  |  |  |  |  |  |  |  |
| 1. YC Anxiety | **.28** | **.24** | **.35** | **.28** | **.38** |  |  |  |  |  |  |  |  |  |  |  |
| 1. Y3 Depression | **.25** | **.26** | **.21** | **.58** | **.30** | **.24** |  |  |  |  |  |  |  |  |  |  |
| 1. Y7 Depression | **.28** | **.38** | **.27** | **.36** | **.58** | **.23** | **.33** |  |  |  |  |  |  |  |  |  |
| 1. YC Depression | **.22** | **.24** | **.36** | **.28** | **.34** | **.58** | **.23** | **.35** |  |  |  |  |  |  |  |  |
| 1. Y6 SAT | **-.15** | **-.21** | **-.14** | **-.20** | **-.20** | -.07** | **-.15** | **-.27** | **-.12** |  |  |  |  |  |  |  |
| 1. Y7 SAT | **-.15** | **-.25** | **-.14** | **-.19** | **-.27** | -.08** | **-.16** | **-.38** | **-.15** | **.29** |  |  |  |  |  |  |
| 1. YC SAT | **-.14** | **-.18** | **-.25** | **-.13** | **-.18** | **-.19** | -.12** | **-.27** | **-.35** | **.28** | **.26** |  |  |  |  |  |
| 1. Financial worries | .12** | .07** | **.11** | .11** | **.11** | **.24** | .01 | .06* | **.17** | -.05* | -.04 | **-.14** |  |  |  |  |
| 1. Health worries | .08* | **.11** | **.15** | .09* | **.10** | **.30** | .04 | .02 | **.13** | .01 | -.00 | -.01 | **.15** |  |  |  |
| 1. Increase discrimination | .06 | -.01 | .01 | .01 | -.04 | .03 | -.02 | -.01 | .02 | .03 | -.01 | .00 | .07** | .01 |  |  |
| 1. Stable discrimination | .06 | .07** | .02 | -.04 | .05* | .02 | -.00 | .02 | .01 | -.02 | -.03 | -.04 | .04 | .01 | **-.09** |  |
| 1. Contact Covid-19 | .03 | -.02 | .04 | -.02 | -.04 | .05* | -.02 | -.01 | .05* | -.01 | .04 | .04 | .00 | .02 | .01 | **-.08** |

*Notes.* Correlations in bold significant at *p* < .001; * *p* < .05, ** *p* < .01.

*Table A2.2.* Correlations main variables: FSU / CEE

|  | 1 | 2 | 3 | 4 | 5 | 6 | 7 | 8 | 9 | 10 | 11 | 12 | 13 | 14 | 15 | 16 |
| --- | --- | --- | --- | --- | --- | --- | --- | --- | --- | --- | --- | --- | --- | --- | --- | --- |
| 1. Y5 PSC |  |  |  |  |  |  |  |  |  |  |  |  |  |  |  |  |
| 1. Y7 PSC | **.49** |  |  |  |  |  |  |  |  |  |  |  |  |  |  |  |
| 1. YC PSC | **.41** | **.51** |  |  |  |  |  |  |  |  |  |  |  |  |  |  |
| 1. Y3 Anxiety | **.27** | **.23** | **.22** |  |  |  |  |  |  |  |  |  |  |  |  |  |
| 1. Y7 Anxiety | **.27** | **.43** | **.25** | **.34** |  |  |  |  |  |  |  |  |  |  |  |  |
| 1. YC Anxiety | .18** | **.27** | **.32** | **.26** | **.36** |  |  |  |  |  |  |  |  |  |  |  |
| 1. Y3 Depression | **.27** | **.22** | .20** | **.55** | **.35** | .17** |  |  |  |  |  |  |  |  |  |  |
| 1. Y7 Depression | **.27** | **.43** | **.28** | **.30** | **.58** | **.28** | **.43** |  |  |  |  |  |  |  |  |  |
| 1. YC Depression | .16** | **.30** | **.33** | .15* | **.34** | **.55** | **.24** | **.38** |  |  |  |  |  |  |  |  |
| 1. Y6 SAT | **-.26** | **-.26** | **-.19** | -.17** | **-.27** | **-.15** | **-.27** | **-.31** | **-.17** |  |  |  |  |  |  |  |
| 1. Y7 SAT | -.17** | **-.31** | **-.20** | -.11 | **-.28** | -.14** | **-.23** | **-.44** | **-.19** | **.36** |  |  |  |  |  |  |
| 1. YC SAT | -.12* | **-.22** | **-.19** | -.02 | **-.18** | **-.15** | -.14* | **-.21** | **-.26** | **.23** | **.26** |  |  |  |  |  |
| 1. Financial worries | .10 | **.16** | **.16** | .16** | .13** | **.34** | **.22** | .14** | **.20** | -.08* | -.09* | -.14** |  |  |  |  |
| 1. Health worries | .00 | .08* | .11** | .04 | .09* | **.38** | -.07 | .01 | **.15** | -.07 | .00 | -.04 | **.21** |  |  |  |
| 1. Increase discrimination | .05 | .02 | -.03 | -.02 | .01 | .07 | -.08 | .00 | .11** | .03 | -.01 | .03 | .01 | .08* |  |  |
| 1. Stable discrimination | -.07 | -.00 | .01 | -.03 | .00 | .07 | -.06 | .07 | .04 | -.01 | -.04 | -.06 | .05 | -.05 | **-.14** |  |
| 1. Contact Covid-19 | -.13* | -.06 | .02 | -.11 | -.09* | .02 | -.00 | -.08 | .01 | -.01 | .04 | .05 | .06 | -.02 | .01 | **-.14** |

*Notes.* Correlations in bold significant at *p* < .001.

* *p* < .05, ** *p* < .01.

*Table A2.3*. Correlations main variables: Other European / Americas

|  | 1 | 2 | 3 | 4 | 5 | 6 | 7 | 8 | 9 | 10 | 11 | 12 | 13 | 14 | 15 | 16 |
| --- | --- | --- | --- | --- | --- | --- | --- | --- | --- | --- | --- | --- | --- | --- | --- | --- |
| 1. Y5 PSC |  |  |  |  |  |  |  |  |  |  |  |  |  |  |  |  |
| 1. Y7 PSC | **.54** |  |  |  |  |  |  |  |  |  |  |  |  |  |  |  |
| 1. YC PSC | **.48** | **.56** |  |  |  |  |  |  |  |  |  |  |  |  |  |  |
| 1. Y3 Anxiety | **.29** | **.38** | **.21** |  |  |  |  |  |  |  |  |  |  |  |  |  |
| 1. Y7 Anxiety | **.35** | **.41** | **.35** | **.40** |  |  |  |  |  |  |  |  |  |  |  |  |
| 1. YC Anxiety | .25** | **.25** | **.35** | **.32** | **.38** |  |  |  |  |  |  |  |  |  |  |  |
| 1. Y3 Depression | **.31** | **.31** | .10 | **.47** | **.35** | .12 |  |  |  |  |  |  |  |  |  |  |
| 1. Y7 Depression | **.33** | **.37** | **.26** | **.22** | **.60** | **.21** | **.43** |  |  |  |  |  |  |  |  |  |
| 1. YC Depression | **.29** | **.26** | **.32** | **.34** | **.36** | **.57** | **.38** | **.45** |  |  |  |  |  |  |  |  |
| 1. Y6 SAT | -.23** | **-.28** | -.15* | **-.29** | **-.27** | -.14* | -.26** | **-.36** | -.19** |  |  |  |  |  |  |  |
| 1. Y7 SAT | -.18* | **-.30** | -.09 | -.04 | **-.24** | -.06 | -.12 | **-.38** | -.18** | **.37** |  |  |  |  |  |  |
| 1. YC SAT | -.18* | **-.22** | -.18** | -.14 | -.16** | -.19** | -.08 | **-.22** | **-.29** | **.28** | **.34** |  |  |  |  |  |
| 1. Financial worries | .16 | **.25** | .19** | .14 | .17** | **.21** | .12 | .10 | .15** | **-.20** | -.17** | **-.20** |  |  |  |  |
| 1. Health worries | .12 | .16** | .11* | .13 | .07 | **.36** | .12 | .08 | .11 | -.10 | -.10 | -.10 | .15** |  |  |  |
| 1. Increase discrimination | .03 | .08 | .06 | -.05 | .14* | .04 | .01 | .05 | .02 | -.04 | -.07 | -.02 | .01 | -.01 |  |  |
| 1. Stable discrimination | -.11 | .02 | -.01 | .06 | .02 | -.09 | .05 | .02 | -.07 | -.08 | -.04 | -.07 | .06 | .12* | -.15** |  |
| 1. Contact Covid-19 | -.10 | -.01 | -.02 | .01 | .00 | .09 | -.06 | -.04 | .00 | -.02 | .04 | .04 | -.05 | -.03 | .09 | -.10 |

*Notes.* Correlations in bold significant at *p* < .001.

* *p* < .05, ** *p* < .01.

*Table A2.4*. Correlations main variables: Asian

|  | 1 | 2 | 3 | 4 | 5 | 6 | 7 | 8 | 9 | 10 | 11 | 12 | 13 | 14 | 15 | 16 |
| --- | --- | --- | --- | --- | --- | --- | --- | --- | --- | --- | --- | --- | --- | --- | --- | --- |
| 1. Y5 PSC |  |  |  |  |  |  |  |  |  |  |  |  |  |  |  |  |
| 1. Y7 PSC | .48* |  |  |  |  |  |  |  |  |  |  |  |  |  |  |  |
| 1. YC PSC | .55** | **.66** |  |  |  |  |  |  |  |  |  |  |  |  |  |  |
| 1. Y3 Anxiety | .56** | .33 | .27 |  |  |  |  |  |  |  |  |  |  |  |  |  |
| 1. Y7 Anxiety | .41 | .29* | .31** | .58** |  |  |  |  |  |  |  |  |  |  |  |  |
| 1. YC Anxiety | .41* | .36** | .51*** | .48* | **.46** |  |  |  |  |  |  |  |  |  |  |  |
| 1. Y3 Depression | .48* | .29 | .35 | **.73** | .41* | .34 |  |  |  |  |  |  |  |  |  |  |
| 1. Y7 Depression | .11 | .18 | .25* | .35 | **.76** | .26* | .36 |  |  |  |  |  |  |  |  |  |
| 1. YC Depression | .09 | .31* | **.49** | .35 | **.44** | **.61** | .49* | **.51** |  |  |  |  |  |  |  |  |
| 1. Y6 SAT | -.07 | -.08 | -.21 | -.22 | -.27* | -.01 | -.30 | -.25* | -.14 |  |  |  |  |  |  |  |
| 1. Y7 SAT | -.05 | -.19 | -.11 | -.24 | **-.45** | .06 | -.16 | **-.43** | -.15 | **.42** |  |  |  |  |  |  |
| 1. YC SAT | .04 | .05 | -.11 | -.26 | -.37** | -.11 | -.30 | -.33** | -.30* | .35** | **.44** |  |  |  |  |  |
| 1. Financial worries | .47* | -.00 | .07 | .53** | .22 | .25* | .39* | .10 | .22 | -.09 | .01 | -.15 |  |  |  |  |
| 1. Health worries | .06 | -.04 | .17 | .03 | .05 | .15 | .11 | .07 | .19 | -.07 | -.06 | -.26* | .14 |  |  |  |
| 1. Increase discrimination | .06 | .15 | .22* | .31 | .13 | .29** | .14 | .05 | .23* | -.15 | -.04 | -.15 | .02 | .26* |  |  |
| 1. Stable discrimination | .01 | .01 | -.07 | -.18 | .11 | -.06 | -.13 | .20 | -.10 | .11 | -.05 | .11 | -.01 | -.23 | **-.72** |  |
| 1. Contact Covid-19 | -.15 | -.01 | -.15 | -.09 | .08 | -.11 | -.03 | .03 | .05 | -.15 | -.18 | -.06 | -.20 | -.22 | -.00 | -.18 |

*Notes.* Correlations in bold significant at *p* < .001.

* *p* < .05, ** *p* < .01.

*Table A2.5*. Correlations main variables: Turkey / ME / Africa

|  | 1 | 2 | 3 | 4 | 5 | 6 | 7 | 8 | 9 | 10 | 11 | 12 | 13 | 14 | 15 | 16 |
| --- | --- | --- | --- | --- | --- | --- | --- | --- | --- | --- | --- | --- | --- | --- | --- | --- |
| 1. Y5 PSC |  |  |  |  |  |  |  |  |  |  |  |  |  |  |  |  |
| 1. Y7 PSC | **.40** |  |  |  |  |  |  |  |  |  |  |  |  |  |  |  |
| 1. YC PSC | **.32** | **.53** |  |  |  |  |  |  |  |  |  |  |  |  |  |  |
| 1. Y3 Anxiety | .07 | .16* | .18** |  |  |  |  |  |  |  |  |  |  |  |  |  |
| 1. Y7 Anxiety | .20** | **.37** | **.25** | **.36** |  |  |  |  |  |  |  |  |  |  |  |  |
| 1. YC Anxiety | .08 | **.19** | **.26** | .19** | **.28** |  |  |  |  |  |  |  |  |  |  |  |
| 1. Y3 Depression | .14* | .19** | **.25** | **.60** | **.31** | .12 |  |  |  |  |  |  |  |  |  |  |
| 1. Y7 Depression | .18* | **.38** | **.23** | **.28** | **.55** | .14** | **.32** |  |  |  |  |  |  |  |  |  |
| 1. YC Depression | .14* | **.18** | **.18** | **.26** | **.25** | **.58** | .18** | **.29** |  |  |  |  |  |  |  |  |
| 1. Y6 SAT | -.16* | **-.24** | **-.17** | **-.22** | **-.22** | -.05 | **-.26** | **-.27** | -.11* |  |  |  |  |  |  |  |
| 1. Y7 SAT | -.07 | **-.23** | -.09 | -.18** | **-.29** | -.10* | -.20** | **-.38** | **-.19** | **.22** |  |  |  |  |  |  |
| 1. YC SAT | -.07 | -.15** | **-.18** | -.10 | **-.19** | **-.18** | -.05 | **-.17** | **-.23** | **.18** | **.19** |  |  |  |  |  |
| 1. Financial worries | .03 | .08 | .07 | .08 | .12* | **.26** | .01 | .04 | **.17** | -.03 | -.09 | -.11* |  |  |  |  |
| 1. Health worries | .08 | .11* | .12** | .02 | .09 | **.47** | -.02 | -.03 | **.23** | -.01 | .01 | -.05 | **.31** |  |  |  |
| 1. Increase discrimination | .05 | .02 | .11* | .10 | -.00 | .12** | -.01 | -.03 | .15** | -.10* | -.02 | -.07 | .07 | .02 |  |  |
| 1. Stable discrimination | -.06 | -.02 | -.02 | -.01 | -.01 | -.07 | .02 | .02 | -.06 | .03 | .04 | .03 | -.01 | -.03 | **-.58** |  |
| 1. Contact Covid-19 | .03 | -.00 | .04 | -.02 | .03 | .02 | .04 | -.02 | .01 | -.00 | .02 | -.01 | -.05 | .02 | -.06 | .05 |

*Notes.* Correlations in bold significant at *p* < .001.

* *p* < .05, ** *p* < .01.
