# Supplementary material for: Changes in young adults' mental well-being before and during the early stage of the COVID-19 pandemic: disparities between ethnic groups in Germany
Source: Child Adolesc Psychiatry Ment Health. 2021 Nov 23;15:69. doi: 10.1186/s13034-021-00418-x (PMC8609988; doi:10.1186/s13034-021-00418-x)
Supplement: Supplementary file 3 — Additional file 3. Total, direct and indirect effects of minority groups on changes in well-being via pandemic-related stressors. [file 13034_2021_418_MOESM3_ESM.docx]

*Additional file 3*

*Table A3.* Total, direct and indirect effects of minority groups on changes in well-being via pandemic-related stressors.

|  | Psychosomatic complaints | Anxiety | Depression | Life satisfaction |
| --- | --- | --- | --- | --- |
| FSU/CEE via |  |  |  |  |
| Total effect | -0.07 (0.06) | -0.06 (0.06) | -0.02 (0.06) | -0.06 (0.18) |
| Direct effect | -0.06 (0.06) | -0.05 (0.06) | -0.02 (0.06) | -0.03 (0.18) |
| Total indirect | -0.01 (0.01) | -0.00 (0.02) | 0.00 (0.01) | -0.03 (0.02) |
| Specific indirect effects |  |  |  |  |
| Financial worries | 0.00 (0.00) | 0.01 (0.01) | 0.01 (0.01) | -0.01 (0.01) |
| Health worries | 0.00 (0.00) | 0.00 (0.02) | 0.00 (0.01) | 0.00 (0.00) |
| Increase discrimination | 0.00 (0.00) | 0.00 (0.00) | -0.00 (0.00) | 0.00 (0.00) |
| Stable discrimination | 0.00 (0.00) | 0.00 (0.00) | 0.00 (0.00) | -0.01 (0.01) |
| Contact with COVID-19 | -0.01 (0.01) | -0.01 (0.01) | -0.01 (0.00) | -0.01 (0.01) |
| Other European/Americas via | | | | |
| Total effect | -0.11 (0.07) | -0.09 (0.07) | 0.03 (0.08) | 0.09 (0.26) |
| Direct effect | -0.11 (0.07) | -0.10 (0.07) | 0.02 (0.08) | 0.14 (0.26) |
| Total indirect | -0.00 (0.01) | 0.02 (0.02) | 0.01 (0.02) | -0.05 (0.03) |
| Specific indirect effects |  |  |  |  |
| Financial worries | 0.00 (0.00) | 0.02 (0.01) | 0.01 (0.01) | -0.03 (0.02) |
| Health worries | 0.00 (0.00) | 0.00 (0.02) | 0.00 (0.01) | -0.00 (0.01) |
| Increase discrimination | -0.00 (0.00) | -0.01 (0.00) | -0.00 (0.00) | -0.00 (0.00) |
| Stable discrimination | 0.00 (0.00) | 0.00 (0.00) | 0.00 (0.01) | -0.01 (0.02) |
| Contact with COVID-19 | -0.00 (0.01) | -0.00 (0.01) | -0.00 (0.01) | -0.00 (0.01) |
| Asia via |  |  |  |  |
| Total effect | 0.26 (0.14) | 0.17 (0.09) | 0.11 (0.13) | -0.06 (0.26) |
| Direct effect | 0.23 (0.15) | 0.02 (0.12) | -0.07 (0.13) | -0.07 (0.28) |
| Total indirect | 0.03 (0.06) | 0.15 (0.07) * | 0.18 (0.07) * | 0.01 (0.15) |
| Specific indirect effects |  |  |  |  |
| Financial worries | 0.00 (0.00) | 0.01 (0.02) | 0.01 (0.02) | -0.02 (0.03) |
| Health worries | 0.01 (0.01) | 0.08 (0.03) ** | 0.05 (0.02) * | -0.02 (0.03) |
| Increase discrimination | 0.04 (0.06) | 0.06 (0.06) | 0.13 (0.06) * | 0.05 (0.15) |
| Stable discrimination | 0.00 (0.00) | 0.00 (0.00) | -0.00 (0.00) | 0.00 (0.01) |
| Contact with COVID-19 | -0.01 (0.01) | -0.01 (0.01) | -0.01 (0.01) | -0.01 (0.01) |
| Turkey/ME/Africa via | | | | |
| Total effect | -0.07 (0.07) | 0.14 (0.07) * | 0.07 (0.09) | -0.39 (0.24) |
| Direct effect | -0.07 (0.08) | 0.07 (0.07) | 0.00 (0.09) | -0.31 (0.25) |
| Total indirect | 0.00 (0.02) | 0.07 (0.03) * | 0.07 (0.03) * | -0.08 (0.07) |
| Specific indirect effects |  |  |  |  |
| Financial worries | 0.00 (0.00) | 0.01 (0.01) | 0.01 (0.01) | -0.02 (0.02) |
| Health worries | 0.00 (0.01) | 0.06 (0.02) ** | 0.03 (0.01) * | -0.02 (0.02) |
| Increase discrimination | 0.01 (0.01) | 0.01 (0.01) | 0.02 (0.01) | 0.01 (0.03) |
| Stable discrimination | 0.01 (0.01) | -0.00 (0.01) | 0.01 (0.01) | -0.04 (0.04) |
| Contact with COVID-19 | -0.01 (0.01) | -0.01 (0.01) | -0.01 (0.01) | -0.01 (0.02) |

*Notes*. Table shows unstandardized effects and standard errors in parentheses.

** p* < .05, ** *p* < .01, *** *p* < .001.
