# Supplementary material for: Changes in young adults' mental well-being before and during the early stage of the COVID-19 pandemic: disparities between ethnic groups in Germany
Source: Child Adolesc Psychiatry Ment Health. 2021 Nov 23;15:69. doi: 10.1186/s13034-021-00418-x (PMC8609988; doi:10.1186/s13034-021-00418-x)
Supplement: Supplementary file 4 — Additional file 4. Robustness checks for the fixed effects regressions based on respondents with no missing information. [file 13034_2021_418_MOESM4_ESM.docx]

*Additional file 4*

*Table A4.* Robustness checks for the fixed effects regressions based on respondents with no missing information (weighted).

|  | Psychosomatic complaints | Anxiety | Depression | Life satisfaction |
| --- | --- | --- | --- | --- |
| T1 | 0.03 (0.03) | -0.18 (0.03) *** | -0.15 (0.03) *** | 0.56 (0.05) *** |
| TC | -0.06 (0.03) * | -0.40 (0.03) *** | -0.30 (0.03) *** | -0.05 (0.05) |
| T1 x FSU/CEE | 0.12 (0.06) * | -0.03 (0.06) | -0.01 (0.06) | -0.19 (0.12) |
| T1 x Other European/Americas | 0.07 (0.08) | -0.13 (0.08) | -0.07 (0.08) | 0.05 (0.16) |
| T1 x Asia | -0.07 (0.20) | -0.09 (0.18) | -0.27 (0.19) | -0.55 (0.35) |
| T1 x Turkey/ME/Africa | 0.11 (0.08) | -0.11 (0.08) | -0.15 (0.08) | -0.23 (0.16) |
| TC x FSU/CEE | -0.10 (0.06) | -0.08 (0.06) | -0.02 (0.06) | -0.05 (0.12) |
| TC x Other European/Americas | -0.14 (0.08) | -0.03 (0.08) | 0.06 (0.08) | 0.15 (0.16) |
| TC x Asia | 0.34 (0.20) | 0.15 (0.18) | 0.14 (0.19) | 0.08 (0.35) |
| TC x Turkey/ME/Africa | -0.16 (0.08) * | 0.04 (0.08) | -0.10 (0.08) | -0.25 (0.16) |
| Unbalanced panel | n = 1.396  T = 3  N = 4.188 | n = 1.388  T = 3  N = 4.164 | n = 1.382  T = 3  N = 4.146 | n = 3.163  T = 3  N = 9.489 |
|  | *F*(10,2782)=9.51*** | *F*(10,2766)=31.11*** | *F*(10,2754)=18.45*** | *F*(10,6316)=24.16*** |
|  | *R^2^* = .031 | *R^2^* = .096 | *R^2^* = .038 | *R^2^* = .043 |

*Notes.* Table shows unstandardized coefficients and standard errors in parentheses. T2 and German are reference categories. Analyses were conducted in R. Calibrated weights were included. Lowercase n represents the number of individuals included in the analyses. Capital T shows how often an individual was observed. Capital N corresponds to the total number of observations in the pooled model (across time); * *p* < .05, ** *p* < .01, *** *p* < .001
