# Supplementary material for: Changes in young adults' mental well-being before and during the early stage of the COVID-19 pandemic: disparities between ethnic groups in Germany
Source: Child Adolesc Psychiatry Ment Health. 2021 Nov 23;15:69. doi: 10.1186/s13034-021-00418-x (PMC8609988; doi:10.1186/s13034-021-00418-x)
Supplement: Supplementary file 5 — Additional file 5. Robustness checks for the fixed effects regressions including changes in marriage and parental status. [file 13034_2021_418_MOESM5_ESM.docx]

*Additional file 5*

*Table A5.* Robustness checks for the fixed effects regressions including changes in marriage and parental status (weighted).

|  | Psychosomatic complaints | Anxiety | Depression | Life satisfaction |
| --- | --- | --- | --- | --- |
| T1 | 0.06 (0.03) * | -0.21 (0.03) *** | -0.11 (0.03) ** | 0.65 (0.05) *** |
| TC | -0.06 (0.02) *** | -0.40 (0.02) *** | -0.26 (0.02) *** | -0.06 (0.05) |
| T1 x FSU/CEE | 0.11 (0.05) * | -0.02 (0.05) | 0.00 (0.06) | -0.18 (0.12) |
| T1 x Other European/Americas | 0.09 (0.07) | -0.14 (0.07) * | -0.06 (0.07) | 0.03 (0.16) |
| T1 x Asia | -0.14 (0.17) | -0.08 (0.16) | -0.27 (0.17) | -0.57 (0.35) |
| T1 x Turkey/ME/Africa | 0.14 (0.07) * | -0.08 (0.07) | -0.07 (0.08) | -0.14 (0.16) |
| TC x FSU/CEE | -0.09 (0.04) * | -0.05 (0.04) | -0.01 (0.04) | -0.08 (0.12) |
| TC x Other European/Americas | -0.12 (0.05) * | -0.07 (0.06) | 0.03 (0.06) | 0.12 (0.16) |
| TC x Asia | 0.21 (0.12) | 0.18 (0.12) | 0.14 (0.13) | 0.09 (0.35) |
| TC x Turkey/ME/Africa | -0.13 (0.05) * | 0.12 (0.06) * | 0.05 (0.06) | -0.28 (0.16) |
| Married | -0.05 (0.06) | 0.03 (0.06) | -0.03 (0.06) | 0.51 (0.15) *** |
| Married missing | 0.08 (0.11) | 0.36 (0.10) *** | -0.01 (0.11) | 0.27 (0.26) |
| Children | -0.00 (0.06) | 0.02 (0.06) | -0.13 (0.06) * | 0.26 (0.15) |
| Children missing | -0.14 (0.11) | -0.32 (0.10) ** | -0.06 (0.11) | -0.48 (0.27) |
| Unbalanced panel | n = 3.185  T = 2-3  N = 7.766 | n = 3.179  T = 2-3  N = 7.746 | n = 3.169  T = 2-3  N = 7.720 | n = 3.198  T = 2-3  N = 9.559 |
|  | *F*(14,4567)=9.10*** | *F*(14,4553)=56.50*** | *F*(14,4537=22.24*** | *F*(10,6351)=25.08*** |
|  | *R^2^* = .024 | *R^2^* = .138 | *R^2^* = .041 | *R^2^* = .044 |

*Notes.* Table shows unstandardized coefficients and standard errors in parentheses. T2, German, unmarried and no children are reference categories. Analyses were conducted in R. Calibrated weights were included. Lowercase n represents the number of individuals included in the analyses. Capital T shows how often an individual was observed. Capital N corresponds to the total number of observations in the pooled model (across time); * *p* < .05, ** *p* < .01, *** *p* < .001.
