# Supplementary material for: Changes in young adults' mental well-being before and during the early stage of the COVID-19 pandemic: disparities between ethnic groups in Germany
Source: Child Adolesc Psychiatry Ment Health. 2021 Nov 23;15:69. doi: 10.1186/s13034-021-00418-x (PMC8609988; doi:10.1186/s13034-021-00418-x)
Supplement: Supplementary file 6 — Additional file 6. Robustness check for the path analysis using only respondents with complete data on all mental well-being outcomes. [file 13034_2021_418_MOESM6_ESM.docx]

*Additional file 6*

*Table A6.* Robustness check for the path analysis using only respondents with complete data on all mental well-being outcomes (weighted)

|  | Financial worries | Health worries | Increase in discrimination | No change in discrimination | Contact with COVID-19 |
| --- | --- | --- | --- | --- | --- |
| *Minority group → stressors* |  |  |  |  |  |
| FSU/CEE | 0.09 (0.08) | 0.00 (0.07) | -0.00 (0.01) | 0.08 (0.04)* | -0.07 (0.04) |
| Other European/Americas | 0.20 (0.10)^*^ | - 0.00 (0.08) | -0.01 (0.01) | 0.10 (0.05)^*^ | -0.04 (0.05) |
| Asia | 0.06 (0.22) | 0.40 (0.15)^**^ | 0.39 (0.10)^***^ | -0.04 (0.10) | -0.07 (0.07) |
| Turkey/ME/Africa | 0.16 (0.10) | 0.25 (0.09)^**^ | 0.07 (0.02)^**^ | 0.33 (0.04)^***^ | -0.11 (0.04)^**^ |
|  | *R^2^=*.02 ^*^ | *R^2^=*.03 ^*^ | *R^2^=*.09 ^**^ | *R^2^=*.06 ^***^ | *R^2^=*.02 ^***^ |
|  | Psychosomatic complaints | Anxiety | Depression | Life satisfaction |  |
| *Stressors → well-being* |  |  |  |  |  |
| Financial worries | 0.01 (0.02) | 0.10 (0.02)^***^ | 0.08 (0.03)^**^ | -0.17 (0.07)^*^ |  |
| Health worries | 0.01 (0.03) | 0.21 (0.02)^***^ | 0.11 (0.03)^***^ | -0.06 (0.08) |  |
| Increase discrimination | 0.09 (0.15) | 0.18 (0.15) | 0.37 (0.14)^**^ | 0.14 (0.39) |  |
| Stable discrimination | 0.01 (0.04) | -0.00 (0.04) | 0.04 (0.05) | -0.12 (0.15) |  |
| Contact with Covid-19 | 0.12 (0.06)^*^ | 0.11 (0.04)^**^ | 0.09 (0.04)^*^ | 0.11 (0.13) |  |
| *Minority group → well-being* |  |  |  |  |  |
| FSU/CEE | -0.06 (0.06) | -0.07 (0.06) | -0.03 (0.06) | -0.02 (0.18) |  |
| Other European/Americas | -0.11 (0.07) | -0.10 (0.07) | 0.03 (0.08) | 0.15 (0.26) |  |
| Asia | 0.23 (0.15) | 0.05 (0.12) | -0.06 (0.13) | -0.12 (0.28) |  |
| Turkey/ME/Africa | -0.07 (0.08) | 0.07 (0.07) | -0.01 (0.09) | -0.31 (0.25) |  |
|  | *R^2^=*.03 ^**^ | *R^2^=*.08 ^***^ | *R^2^=*.04 ^***^ | *R^2^=*.02 ^*^ |  |

*Notes*. Table shows unstandardized coefficients and standard errors in parentheses; All models control for gender, age, participants’ education, and parental occupational status; ** p* < .05, ** *p* < .01, *** *p* < .001.
